# Protocol for PPP1R15A-inhibited mouse model establishment with subcutaneous B16F1 tumor and single-cell analysis

**DOI:** 10.1016/j.xpro.2023.102616

**Published:** 2023-09-26

**Authors:** Rongjing Wang, Minghui Wang, Siyu Pei, Yuchao Zhang, Shiwei Guo, Wei Guo, Zhenchuan Wu, Hailong Wang, Yizhe Li, Yufei Zhu, Ling-Hua Meng, Jingyu Lang, Gang Jin, Yichuan Xiao, Landian Hu, Xiangyin Kong

**Affiliations:** 1 Shanghai Institute of Nutrition and Health, Chinese Academy of Sciences, CAS Key Laboratory of Tissue Microenvironment and Tumor, Shanghai, China; 2 University of Chinese Academy of Sciences, Shanghai, China; 3 Department of Thoracic Surgical Oncology, Shanghai Lung Cancer Center, Shanghai Chest Hospital, Shanghai Jiao Tong University School of Medicine (SJTUSM), Shanghai 200030, China; 4 Changhai Hospital, Department of Hepatobiliary Pancreatic Surgery, Shanghai, China; 5 ShanghaiTech University, School of Life Science and Technology, Shanghai, China; 6 Division of Anti-tumor Pharmacology, Shanghai Institute of Materia Medica, Chinese Academy of Sciences, Shanghai, China; 7 Anda Biology Medicine Development (Shenzhen) Co., Ltd, Shenzhen, China

**Keywords:** Bioinformatics, Cancer, Immunology

## Abstract

Here, we present a protocol for exploring the effects of PPP1R15A inhibitor, Sephin1, on antitumor immunity of B16F1 subcutaneous tumor in mice. We describe steps for constructing single-cell transcriptome and TCR libraries, sequencing, and using sequencing data for the integration of expression and TCR data. We then detail procedures for gene differentiation, regulon and cell-cell communication analysis, and validation of single-cell analysis results.

For complete details on the use and execution of this protocol, please refer to Wang et al.^1^

## Highlights

• Procedure for integrating single-cell transcriptome and TCR enrichment data
• Effective injection procedure of Sephin1 to suppress the antitumor immunity
• Establishing a platform to validate the effects of Sephin1 on antitumor immunity

## Abstract (editor highlights)

Publisher’s note: Undertaking any experimental protocol requires adherence to local institutional guidelines for laboratory safety and ethics.

## Graphical abstract

**Figure:**
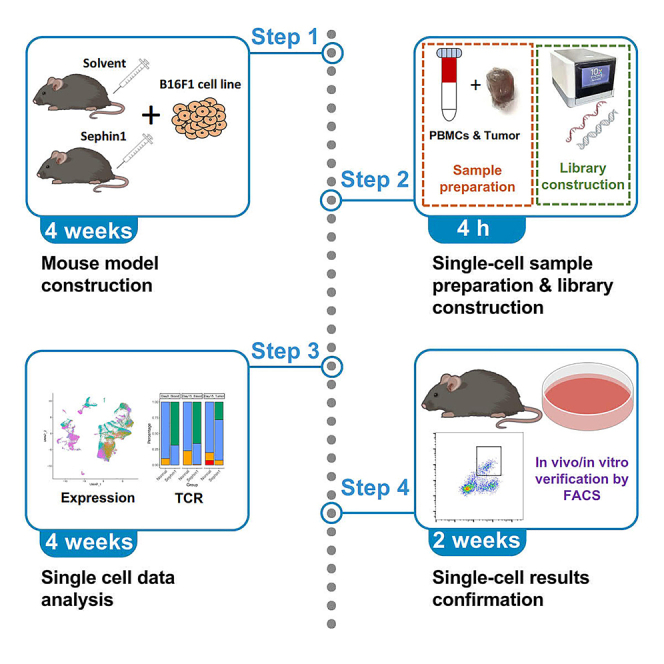

## Before you begin

### Institutional permissions

The mice experiments followed the ethical guidelines prescribed by Experimental Animal Management and Use Committee of Shanghai Institute of Nutrition and Health.

This protocol consists of four parts: mouse model establishment, including Sephin1 injection and B16F1 tumor implantation; single-cell sample preparation and library construction; single-cell data analysis; and single-cell result validation with FACS.

### Reagent preparation

**Timing: 30 min**

1. Preparation of Sephin1 solution.
  a. Warm DMSO and PBS in 37°C water bath for about 15 min.
  b. Put the Sephin1 (50 mg) powder into 625 μL warm DMSO, then into 12.5 mL warm PBS. The final solution is 4 mg/mL Sephin1 and 5% DMSO.
2. Add 2.5 mL DMSO into 47.5 mL PBS for 5% DMSO control solution.

**CRITICAL:** Sephin1 and DMSO solution should be stored at −20°C for no more than 3 months.

### Cell line preparation

**Timing: 7–14 days**

3. Thaw a vial of cryopreserved B16F1 cells in a water bath set to 37°C for 2–3 min.
4. Transfer the cells into a 1.5 mL tube, and then centrifuge the cells at 400 × *g* for 5 min to collect the cells.
5. Transfer the cells into 10 cm dish, and culture them with complete medium containing 10 mL RPMI 1640 medium supplemented with 10% FBS and 1% Pen Strep (Penicillin Streptomycin).
6. Passage the cells every other day with about 4–6 $\times 10^{6}$ cells in each dish.
  a. Digest the cells in each dish with 2 mL TrypLE for 1 min at 37°C.
  b. Terminate the digestion with equal volume (2 mL for each dish) of complete medium.
  c. Centrifuge at 400 × *g* for 5 min and collect the cells. Passage the cell at a ratio of about 1:3 to 1:4.

## Key resources table

**Table:** 

REAGENT or RESOURCE	SOURCE	IDENTIFIER
**Antibodies**		

PE Hamster Anti-Mouse CD3e (dilution: 1:200)	BD Biosciences	553064
FITC anti-mouse TCR β chain Antibody (dilution: 1:200)	BioLegend	109205
PE/Cyanine7 anti-mouse/human CD11b Antibody (dilution: 1:200)	BioLegend	101215
PerCP anti-mouse F4/80 Antibody (dilution: 1:200)	BioLegend	123125
PE anti-mouse CD45 Antibody (dilution: 1:200)	BioLegend	103105
FITC anti-mouse CD45 Recombinant Antibody (dilution: 1:200)	BioLegend	157607
Brilliant Violet 605 anti-mouse CD4 Antibody (dilution: 1:200)	BioLegend	100548
Brilliant Violet 421 anti-mouse FOXP3 Antibody (dilution: 1:200)	BioLegend	126419
PerCP/Cyanine 5.5-conjugated anti-mouse CD8a antibody (dilution: 1:200)	BioLegend	100733
PE-conjugated anti-mouse IFN-γ antibody (dilution: 1:200)	BioLegend	163503
InVivoMAb anti-mouse CD3ε (dilution: 1:200)	BioXCell	BE0001-1-5MG
InVivoMAb anti-mouse CD28 (dilution: 1:200)	BioXCell	BE0015-1-5MG
CD45 Monoclonal Antibody (30-F11), PE, eBioscience (dilution: 1:200)	Thermo Fisher	12-0451-83
CD8a Monoclonal Antibody (53-6.7), PE-Cyanine7, eBioscience (dilution: 1:200)	Thermo Fisher	25-0081-81
NK1.1 Monoclonal Antibody (PK136), eFluor 450, eBioscience (dilution: 1:200)	Thermo Fisher	48-5941-80
CD279 (PD-1) Monoclonal Antibody (J43), APC, eBioscience (dilution: 1:200)	Thermo Fisher	17-9985-82

**Chemicals, peptides, and recombinant proteins**		

Ficoll-Paque PREMIUM	Amersham/GE	17544602
Sephin1	APExBIO	A8708-50
FBS	Biological Industries	04-001-1A
RPMI 1640 medium	Gibco	11875093
Pen Strep	Gibco	15140122
Corning Sterile Cell Strainers, 100 μm	Corning 431752	15380801
MACS® SmartStrainers (30 μm), 4 × 25 pcs	Miltenyi/MACS	130-110-915
AO/PI	Nexcelom Bioscience	CS2-0106-5mL
Propidium iodide solution	Nexcelom Bioscience	CS1-0109-5mL
IL2, mouse	Novoprotein	CK24
IL7, mouse	Novoprotein	CC73
ACK Lysing Buffer	Thermo Fisher	A1049201
TrypLE Express	Thermo Fisher	12604021
0.5 M EDTA, pH8.0	Beyotime	ST066

**Critical commercial assays**		

Chromium Next GEM Single Cell 5′ Library & Gel Bead Kit v1.1	10× Genomics	PN-1000165
Chromium Single Cell 5′ Library Construction Kit	10× Genomics	PN-1000020
Chromium Single Cell V(D)J Enrichment Kit	10× Genomics	PN-1000071
Chromium Next GEM Chip G Single Cell Kit	10× Genomics	PN-1000120
Single Index Kit T Set A	10× Genomics	PN-1000213
BD Cytofix/Cytoperm Fixation/Permeablization Kit	BD Biosciences	554714
Cell Activation Cocktail with Brefeldin A	BioLegend	423303
MojoSort Mouse CD8 T Cell Isolation Kit	BioLegend	480035
CFSE Cell Division Tracker Kit	BioLegend	423801
Tumor Dissociation Kit, mouse	Miltenyi/MACS	130-096-730
LIVE/DEAD Fixable Violet Dead Cell Stain Kit, for 405 nm excitation	Thermo Fisher	L34963
LIVE/DEAD Fixable Near IR (780) Viability Kit, for 633 nm excitation	Thermo Fisher	L34994

**Deposited data**		

Raw and analyzed data	This paper	GEO: GSE220656

**Experimental models: Organisms/strains**		

C57BL/6Slac, mus musculus, wild type, 6–8 weeks, male	SLAC LABORATORY ANIMAL	NA

**Software and algorithms**		

CellRanger	10× Genomics	Version 6.0.0
Seurat	https://satijalab.org/seurat/index.html	Version 3.2.3
scRepertoire	http://www.bioconductor.org/packages/release/bioc/html/scRepertoire.html	Version 1.2.1
Python	https://www.python.org/	Version 2.7.5
R	https://cran.r-project.org/	Version 3.3.5
SCENIC	https://github.com/aertslab/SCENIC	Version 1.1.2–01
GENIE3	http://www.bioconductor.org/packages/release/bioc/html/GENIE3.html	Version 1.8.0
GSVA	http://www.bioconductor.org/packages/release/bioc/html/GSVA.html	Version 1.30.0
fgsea	http://www.bioconductor.org/packages/release/bioc/html/fgsea.html	Version 1.8.0
ggplot2	https://cran.r-project.org/	Version 3.3.5
ggpubr	https://cran.r-project.org/	Version 0.4.0
pheatmap	https://cran.r-project.org/	Version 1.0.12
ComplexHeatmap	https://cran.r-project.org/	Version 2.8.0
CellChat	https://github.com/sqjin/CellChat	Version 1.0.0

**Other**		

Original code	This paper	https://github.com/LisaWang2022/B16F1-mouse-single-cell-analysis
NovaSeq6000 System	Illumina	N/A
CytoFLEX LX	Beckman Coulter	C04312

## Materials and equipment

***Note:*** recipes with ≥3 ingredients should be presented as tables.

**Table:** 5% DMSO control solution

Reagent	Final concentration	Amount
DMSO	5%	2.5 mL
1× PBS	N/A	47.5 mL
**Total**	**N/A**	**50 mL**

Prepare in advance and store at −20°C.

**Table:** 4 mg/mL Sephin1 solution

Reagent	Final concentration	Amount
Sephin1 powder	4 mg/mL	50 mg
DMSO	5%	0.625 mL
1× PBS	N/A	Add to 12.5 mL
**Total**	**N/A**	**12.5 mL**

Prepare in advance and store at −20°C for less than 2 months.

**Table:** RPMI 1640 medium with 10% FBS

Reagent	Final concentration	Amount
Pen Strep	1%	5 mL
FBS	10%	50 mL
RPMI 1640	N/A	445 mL
**Total**	**N/A**	**500 mL**

Prepare in advance and store at 4°C.

**Table:** 5 mg/mL anti-mouse CD3ε stock solution

Reagent	Final concentration	Amount
anti-mouse CD3ε	5 mg/mL	5 mg
ddH2O	N/A	1 mL
**Total**	**N/A**	**1 mL**

Prepare in advance. Storage at −20°C for less than two weeks and −80°C for two weeks -- 6 months.

**Table:** 5 mg/mL anti-mouse CD28 stock solution

Reagent	Final concentration	Amount
anti-mouse CD28	5 mg/mL	5 mg
ddH2O	N/A	1 mL
**Total**	**N/A**	**1 mL**

Prepare in advance. Storage at −20°C for less than two weeks and −80°C for two weeks -- 6 months.

**Table:** Anti-mouse CD3ε-CD28 solution

Reagent	Final concentration	Amount
anti-mouse CD3ε stock solution	1 μg/mL	2 μL
anti-mouse CD28 stock solution	1 μg/mL	2 μL
1× PBS	N/A	10 mL
**Total**	**N/A**	**10 mL**

Prepare immediately before use.

## Step-by-step method details

### Mouse model establishment

**Timing: 4 weeks**

This procedure describes the construction of mouse model, including the chemical injection and tumor cell implantation. Mice used are six- to eight-week-old male C57BL/6 mice.

1. Separate the C57BL/6 mice into two groups randomly: the control group and the Sephin1 group. Each group should contain about 8–12 mice.
2. Chemical injection.
  a. Inject the Sephin1 group mice with 100 μL Sephin1 solution intraperitoneally, while the control group with equal volume of control solution.
  b. Inject both groups three times per week for two weeks (Day 1, 3, 5 in each week).
3. Tumor implantation. Proceed with tumor implantation two days after the last Sephin 1 pre-treatment.
  a. Collect cultured B16F1 cells with trypsinization as described above.
  b. Resuspend the cells with PBS at a concentration of $2.4\times 10^{6}$/mL. Then measure cell concentration and viability with AO/PI using the equipment Cellometer K2.
  c. Inject each mouse with 125 μL of cell suspension subcutaneously at the left side of mouse abdomen for a total of $3\times 10^{5}$ living cells per mouse.
  d. Measure the tumors are measured every 2–3 days for a total of 15 days post-injection.
    i. Measure the shortest and longest tumor diameters (d and D) with a Vernier caliper every one or two days.
    ii. Calculate the tumor volume (V) as: $V=d^{2}\times D/2$.

**CRITICAL:** Keep the prepared B16F1 cells at 4°C before implantation implant the cells within 30 min after preparation.

### Single-cell sample preparation & library construction

**Timing: 4 h**

This procedure describes the single-cell sample preparation process, including the separation of mouse peripheral blood mononuclear cells (PBMCs) and tumor immune cells.

**Figure 5:**
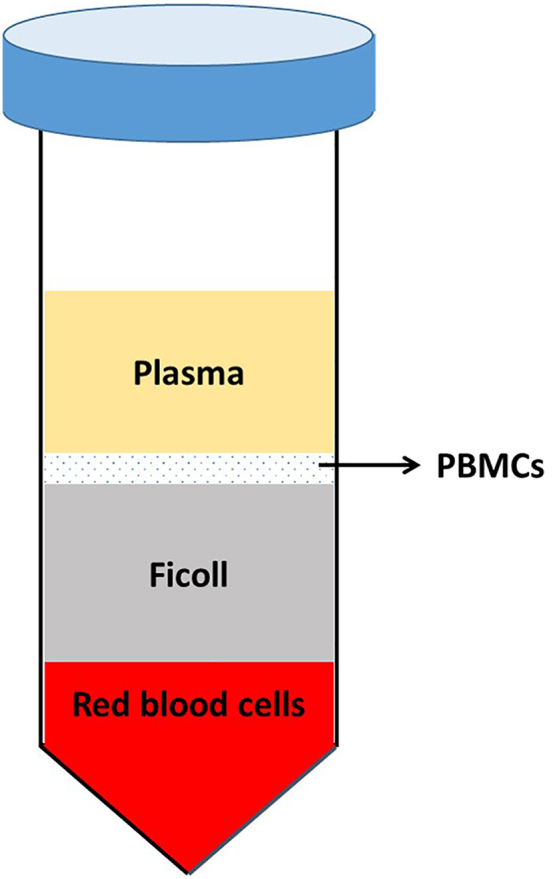
Layer distribution of Ficoll-separated peripheral blood sample
  f. Stain the cells with AO/PI and quantify the cells using the machine Cellometer K2 (Nexcelom Bioscience).
    ***Note:*** The hemocytometer and trypan blue can also be used for cell calculation as an alternative. The percentage of living cells should be more than 70% and the number of living cells should be larger than $2\times 10^{5}$ to ensure experimental success.
5. Mouse tumor immune cell separation. Collect the tumors from mice 15 days post-injection. Choose two mice for tumor immune cells separation in each group. Measure the tumor weight before the immune cell separation procedure.
  a. Collect the tumor tissues and cut them into small pieces (approximately 1–2 mm^3^).
  b. Digest the tumor tissues with the mouse tumor dissociation kit (Miltenyi/MACS, 130-096-730), and then filter the prepared cells with the 30 μm MACS SmarterStrainer.
  c. Incubate the cells with mouse CD45-specific antibody (BioLegend, 157607) for 30 min and then incubate with propidium iodide solution with the volume ratio of 1:100 immediately before FACS cell sorting (Nexcelom Bioscience, CS1-0109-5mL).
  d. Sort the cells on a BD SORP FACSAria with standard procedure to capture CD45-positive and living cells.
6. Use the isolated single-cell suspensions for 10× library construction following standard procedures. The library construction kits include the Chromium Next GEM Single Cell 5′ Library & Gel Bead Kit v1.1 (16 rxns, PN-1000165), Chromium Single Cell 5′ Library Construction Kit (16 rxns, PN-1000020), Chromium Single Cell V(D)J Enrichment Kit (Mouse T cell, 96 rxns, PN-1000071), Chromium Next GEM Chip G Single Cell Kit (48 rxns, PN-1000120) and Single Index Kit T Set A (96 rxns, PN-1000213).
7. Sequence all samples on an Illumina NovaSeq PE150 platform.

***Note:*** All the gene expression libraries are sequenced with a data size of 270 millions of clusters. The data size of each TCR-enriched library is 34 millions of clusters. In addition, the average number of reads per cell is not less than 30 M in the expression data and not less than 15 M in the TCR-enriched data.

4. Mouse PBMCs separation. Collect the blood samples from two time points, the day of tumor cell implantation (Day 0) and 15 days after implantation (Day 15). Then isolate PBMCs from two mice in each group at each time point. Collect PBMCs with density gradient separation method.
  a. Collect the peripheral blood samples from the mouse eyes. About 100–200 μL of samples can be collected from each mouse.
  b. Mix each sample with 200 μL EDTA,. Then add an equal volume of PBS of the blood-EDTA mixture.
  c. Measure the total volume of blood sample, EDTA and PBS. Then add the mixture onto the surface of an equal volume of Ficoll-Paque PREMIUM in 15 mL centrifuge tube.
  d. Centrifuge at 18°C–20°C with 400 × *g* for 20 min with an acceleration of 5 and a deceleration of 3.
  e. Carefully remove the tubes from the centrifuge to avoid disturbing the layers. PBMCs are enriched at the interphase fluffy white layer (Figure 5). Then collect the mononuclear cells carefully without pipetting the adjacent plasma and Ficoll-Paque PREMIUM fluids.
    ***Note:*** If needed, remove the red blood cells by resuspending the cells in ACK lysing buffer and incubating for 3 min at room temperature. Add 1–2 mL of ACK lysing buffer into each sample. After three minutes, add 10 mL PBS into each tube. Then centrifuge the cells with 400 × *g*, 4°C for 5–10 min and wash cells once with PBS.

### Single-cell data analysis

**Timing: 4 weeks**

This procedure describes the bioinformatics analysis of the single-cell data obtained from the last procedure, including raw data processing, data integration, regulon analysis, differentially expressed genes analysis and cell-cell communication analysis. All the steps are completed in Linux, R and Python platforms. Detailed code used in this protocol can be found at this link: https://github.com/LisaWang2022/B16F1-mouse-single-cell-analysis.

8. CellRanger analysis of the single-cell expression data and TCR enrichment data. This procedure is completed with CellRanger version 6.0.0.
  a. CellRanger process of single-cell expression data. Complete this step with the CellRanger function “cellranger count”. Set the local cores as 12 and the expect cells to 8000. Download the reference dataset from the 10× Genomics website: https://cf.10xgenomics.com/supp/cell-exp/refdata-gex-mm10-2020-A.tar.gz.
    ***Note:*** This step is performed on Python.
    #!/usr/bin/python
    #-∗-coding:UTF-8-∗-
    import os,re
    filelist=os.listdir("/data2/project/sephin_mouse_data/")
    def dataprocess(sample,data):
     datadir="/data2/project/sephin_mouse_data/"
     os.chdir(datadir)
     samlist1=[]
     samlist2=[]
     for da in data:
      if re.match(sample,da) and re.search('.fq.gz',da):
       if re.search('_1',da):
        samlist1.append(da)
       elif re.search('_2',da):
        samlist2.append(da)
     samline1=' '.join(samlist1)
     samline2=' '.join(samlist2)
     os.system('cat '+samline1+' > processed_fastqs/'+sample+'_S1_L001_R1_001.fastq.gz')
     os.system('cat '+samline2+' > processed_fastqs/'+sample+'_S1_L001_R2_001.fastq.gz')
     os.chdir('/home/wangrj/wangrj/wangrj/MOUSE_SEPHIN1_RESULTS')
     os.system('/home/wangrj/wangrj/wangrj/softwares/cellranger-6.0.0/cellranger count --localcores=12 --id='+sample+'_output --transcriptome=/data1/database/refdata-cellranger-mm10-1.2.0 --fastqs=/data2/project/sephin_mouse_data/processed_fastqs/ --sample='+sample+' --expect-cells=8000')
    #main
    samplelist=['BN1d0V5','BN2d0V5','BS1d0V5','BS2d0V5','TN1d15V5','TN2d15V5','TS1d15V5','TS2d15V5','BN1d15V5','BN2d15V5','BS1d15V5','BS2d15V5']
    for sa in samplelist:
     dataprocess(sa,filelist)
  b. CellRanger process of TCR enrichment data. Complete this step with the CellRanger function “cellranger vdj”. Set the localcores to 12. Download the reference data from this website: https://cf.10xgenomics.com/supp/cell-vdj/refdata-cellranger-vdj-GRCm38-alts-ensembl-7.0.0.tar.gz.
    ***Note:*** This step is performed on Python.
    #!/usr/bin/python
    #-∗-coding:UTF-8-∗-
    import os,re
    filelist=os.listdir("/data2/project/sephin_mouse_data/")
    def dataprocess(sample,data):
     datadir="/data2/project/sephin_mouse_data/"
     os.chdir(datadir)
     samlist1=[]
     samlist2=[]
     for da in data:
      if re.match(sample,da) and re.search('.fq.gz',da):
       if re.search('_1',da):
        samlist1.append(da)
       elif re.search('_2',da):
        samlist2.append(da)
     samline1=' '.join(samlist1)
     samline2=' '.join(samlist2)
     os.system('cat '+samline1+' > processed_fastqs/'+sample+'_S1_L001_R1_001.fastq.gz')
     os.system('cat '+samline2+' > processed_fastqs/'+sample+'_S1_L001_R2_001.fastq.gz')
     os.chdir('/home/wangrj/wangrj/wangrj/MOUSE_SEPHIN1_RESULTS')
     os.system('/home/wangrj/wangrj/wangrj/softwares/cellranger-6.0.0/cellranger count --localcores=12 --id='+sample+'_output --transcriptome=/data1/database/refdata-cellranger-mm10-1.2.0 --fastqs=/data2/project/sephin_mouse_data/processed_fastqs/ --sample='+sample+' --expect-cells=8000')
    #main
    samplelist=['BN1d0V5','BN2d0V5','BS1d0V5','BS2d0V5','TN1d15V5','TN2d15V5','TS1d15V5','TS2d15V5','BN1d15V5','BN2d15V5','BS1d15V5','BS2d15V5']
    for sa in samplelist:
     dataprocess(sa,filelist)
9. Integration of different data. Include the integration of different samples in expression data and TCR data and the integration of expression data and TCR enrichment data.
  a. Integration of all single-cell expression data.
    ***Note:*** This step is performed on R.
    i. First, load the packages used for analysis afterwords. The single-cell expression data are imported into R and integrated with Seurat (version 3.2.3).
      library(Seurat)
      library(cowplot)
      library(future)
      library(Matrix)
      options(future.globals.maxSize = 1000000 ∗ 1024ˆ2)
    ii. Second, load each data and pre-process each sample. To minimize information loss and filter out low-quality and duplicated cells at the same time, genes expressed in at least 2 cells are kept, and cells with more than 100 but less than 4000 genes are kept.
      filelist<-c("BN1d0V5","BN2d0V5","BS1d0V5","BS2d0V5","BN1d15V5","BN2d15V5","BS1d15V5","BS2d15V5","TN1d15V5","TN2d15V5","TS1d15V5","TS2d15V5")
      namelist<-c("Day0_Blood_Normal_1","Day0_Blood_Normal_2","Day0_Blood_Sehpin1_1","Day0_Blood_Sephin1_2","Day15_Blood_Normal_1","Day15_Blood_Normal_2","Day15_Blood_Sephin1_1","Day15_Blood_Sephin1_2","Day15_Tumor_Normal_1","Day15_Tumor_Normal_2","Day15_Tumor_Sehpin1_1","Day15_Tumor_Sephin1_2")
      mouse.data<-list()
      for(i in 1:length(filelist)){
      fl=filelist[i]
      sname=paste0("/storage/work/wangrj/MOUSE_SEPHIN1_RESULTS/Exp/",fl,"_output/outs/filtered_feature_bc_matrix/")
       sample.raw<-Read10X(sname)
       sample<-CreateSeuratObject(counts=sample.raw,project="MOUSE_SEPHIN1",min.cells=2)
       sample$COMPARE<-namelist[i]
      gname<-gsub("_1","",namelist[i])
      gname<-gsub("_2","",gname)
      sample$SAMPLE<-gname
       sample<-subset(sample,subset=nFeature_RNA>100)
       sample<-NormalizeData(sample,verbose=FALSE)
       sample<-FindVariableFeatures(sample,selection.method="vst",nfeatures=4000)
       mouse.data<-c(mouse.data,list(sample))
      }
      names(mouse.data)<-namelist
    iii. Third, integration the expression data and perform dimensionality reduction and cluster finding analysis.
      plan("multiprocess", workers = 8)
      mouse.anchors<-FindIntegrationAnchors(object.list=mouse.data,dims=1:40)
      mouse.data<-IntegrateData(anchorset=mouse.anchors,dims=1:40)
      DefaultAssay(mouse.data)<-"RNA"
      mouse.data<-FindVariableFeatures(mouse.data)
      mouse.data$percent.mt<-PercentageFeatureSet(mouse.data,pattern="ˆmt-")
      mouse.data<-ScaleData(mouse.data,vars.to.regress = "percent.mt")
      mouse.data<-RunPCA(mouse.data,npcs=100)
      mouse.data <- FindNeighbors(mouse.data, reduction = "pca", dims = 1:50, nn.eps = 0.5)
      mouse.data <- FindClusters(mouse.data, resolution = 3, n.start = 10)
      mouse.data<-RunUMAP(mouse.data,reduction="pca",dims=1:50)
      mouse.data<-RunTSNE(mouse.data,reduction="pca",dims=1:50)
      saveRDS(mouse.data,"mouse_sephin1_all_combined_norm_by_mt.rds")
  b. Integration of all TCR enrichment data.
    ***Note:*** This step is performed on R.
    i. Analyze and integrate the TCR contig matrix using scRepertoire (version 1.2.1).^2^
    ii. Integrate with the gene expression data.
    iii. Separate and annotate TCRs by their distribution in one sample.
      ***Note:*** If the percentage of the clone number of one clonotype in all clones of the sample was between 0.1 and 1, the clonotype was classified as “hyperexpanded”; if the percentage was between 0.01 and 0.1, the classification was “large”. “Medium” was used to denote a percentage between 0.001 and 0.01, and “small” indicated a percentage between $10^{-4}$ and 0.001.
      library(scRepertoire)
      library(stringr)
      library(ggsci)
      csvlist<-c("BN1d0.csv","BN2d0.csv","BS1d0.csv","BS2d0.csv","BN1d15.csv","BN2d15.csv","BS1d15.csv","BS2d15.csv","TN1d15.csv","TN2d15.csv","TS1d15.csv","TS2d15.csv")
      contig_list<-list()
      for(cl in csvlist){
       filename<-paste0("/storage/work/wangrj/MOUSE_SEPHIN1_RESULTS/VDJ_analysis/immunarch_files/TCR/",cl)
       csvfile<-read.csv(filename,stringsAsFactors=F)
       csvfilter<-csvfile[,c('barcode','is_cell','contig_id','high_confidence','length','chain',
          'v_gene','d_gene','j_gene','c_gene','full_length','productive','cdr3',
          'cdr3_nt','reads','umis','raw_clonotype_id','raw_consensus_id')]
       contig_list<-c(contig_list,list(csvfilter))
      }
      combined <- combineTCR(contig_list,samples=c("Day0_Blood","Day0_Blood","Day0_Blood","Day0_Blood","Day15_Blood","Day15_Blood","Day15_Blood","Day15_Blood","Day15_Tumor","Day15_Tumor","Day15_Tumor","Day15_Tumor"),ID=c("Normal","Normal","Sephin1","Sephin1","Normal","Normal","Sephin1","Sephin1","Normal","Normal","Sephin1","Sephin1"),cells="T-AB")
  c. Integration of expression data and TCR enrichment data.
    ***Note:*** This step is performed on Python and R. Plots of different TCR types are made with ggplot2 (version 3.3.5).
    i. First, calculate the TCR clonotype frequency by scRepertoire and save the results to csv file. This step is completed by R.
      library(scRepertoire)
      library(stringr)
      library(ggsci)
      theCall <- function(x) {
       if (x == "gene") {
        x <- "CTgene"
       } else if(x == "nt") {
        x <- "CTnt"
       } else if (x == "aa") {
        x <- "CTaa"
       } else if (x == "gene+nt") {
        x <- "CTstrict"
       }
       return(x)
      }
      clonoTypes<-c(None = 0, Rare = 1e-04, Small = 0.001, Medium = 0.01,
         Large = 0.1, Hyperexpanded = 1)
      cloneCall<-theCall("gene")
      df1<-lapply(combined,"[[",cloneCall)
      df2<-lapply(combined,"[[","barcode")
      data_out<-c()
      for(i in 1:length(df1)){
       Barcode<-df2[[i]]
       CTgene<-df1[[i]]
       datasub<-data.frame(Barcode,CTgene)
       datasub$Num<-i
       data_out<-rbind(data_out,datasub)
      }
      write.csv(data_out,"VDJ_analysis/data_to_match.csv",quote=F,row.names=F)
    ii. Second, make the csv file containing the TCR type result that can be mapped on the expression data. This step is completed on Python.
      #!/usr/bin/python
      #-∗-coding:UTF-8-∗-
      def cal_var_type(number):
       TYPE='None'
       if number==0:
        TYPE='None'
       elif number<=0.0001:
        TYPE='Rare'
       elif number>0.0001 and number<=0.001:
        TYPE='Small'
       elif number>0.001 and number<=0.01:
        TYPE='Medium'
       elif number>0.01 and number<=0.1:
        TYPE='Large'
       else:
        TYPE='Hyperexpanded'
       return TYPE
      filename=open('data_to_match.csv').readlines()
      filename.pop(0)
      #barfile=open('../mouse_cluster_by_lym_detailed.csv').readlines()
      barfile=open('../data_reanalysis/mouse_cluster.csv').readlines()
      barfile.pop(0)
      barcode=[]
      for bf in barfile:
       bfline=bf.strip().split(',')
       barcode.append(bfline[0])
      tcr_result=[]
      for i in range(12):
       tsub=[]
       for fn in filename:
        fnline=fn.strip().split(',')
        tcr_bar=fnline[0]
        tcr_type=fnline[1]
        num=int(fnline[2])-1
        if num==i:
         tsub.append([tcr_bar,tcr_type])
        tcr_result.append(tsub)
      tcr_final={}
      for i in range(12):
       sub=tcr_result[i]
       barsub=[]
       typesub=[]
       for j in range(len(sub)):
        barsub.append(sub[j][0])
        typesub.append(sub[j][1])
       typeeach=list(set(typesub))
       type_per={}
       for te in typeeach:
        per=float(typesub.count(te))/len(typesub)
        type_per[te]=per
       for k in range(len(barsub)):
        barline=barsub[k].split('_')
        barfinal=barline[3]+'_'+barline[4]
        perfinal=type_per[typesub[k]]
        typefinal=cal_var_type(perfinal)
        per_type=str(perfinal)+','+typefinal
        tcr_final[barfinal]=per_type
      outfile=open('../data_reanalysis/matched_tcr_result.csv','w+')
      outfile.write('Barcode,Percentage,Var_type\n')
      for bc in barcode:
       if bc in tcr_final:
        outline=bc+','+tcr_final[bc]+'\n'
       else:
        outline=bc+',0,None\n'
       outfile.write(outline)
      outfile.close()
      tcr_specific=open('tcr_specific_cells.csv','w+')
      for tf in tcr_final:
       if tf in barcode:
        continue
       else:
        tcr_specific.write(tf+'\n')
      tcr_specific.close()
    iii. Third, mapping the TCR type results to the Seurat object containing the expression data, and make the DimPlot graphs and calculate the TCR type specific genes.
      library(scRepertoire)
      library(stringr)
      library(ggsci)
      Clonotype<-read.csv("matched_tcr_result.csv",header=T)
      mouse.data$TCR_type<-Clonotype$Var_type
      pdf("cluster_by_tcr_type_graph.pdf",height=5,width=7)
      DimPlot(mouse.data,group.by="TCR_type",cols=c("red","orange","blue","grey","green"))
      dev.off()
      pdf("cluster_by_tcr_type_graph_splitted.pdf",height=10,width=9)
      DimPlot(mouse.data,split.by="SAMPLE",group.by="TCR_type",cols=c("red","orange","blue","grey","green"),ncol=2)
      dev.off()
      Idents(mouse.data)<-mouse.data$TCR_type
      tcr_all_markers<-FindAllMarkers(mouse.data,min.pct=0.25)
      write.csv(tcr_all_markers,"tcr_all_markers.csv",quote=F)
10. Regulon analysis with Single-Cell Regulatory Network Inference and Clustering (SCENIC).
  SCENIC (version 1.1.2-01)^3^ analysis is performed to analyze the activity of important transcription factors and their related genes.
  a. Randomly select 5000 cells from all 12 samples to identify the coexpression network with higher activities using GENIE3 (version 1.8.0).
  b. Perform SCENIC analysis on all cells, and filter specific regulons from the coexpression network.
  c. Calculate the activities of different regulons in different sample types and cell types.
  d. Calculate the regulon specificity score (RSS) of each sample 4 in order to identify the sample-specific regulons of each sample type.^4^
    library(SCENIC)
    library(Seurat)
    library(GENIE3)
    library(AUCell)
    library(foreach)
    library(RcisTarget)
    library(pheatmap)
    library(ComplexHeatmap)
    library(dplyr)
    counts<-data@assays$RNA@counts
    counts<-as.matrix(counts)
    Gene<-rownames(counts)
    countsnew<-cbind(Gene,counts)
    cell_type<-data$Cluster_name
    Cell<-rownames(data@meta.data)
    meta<-data.frame(Cell,cell_type)
    #SCENIC
    Sample<-data$SAMPLE
    Cluster<-data$seurat_clusters
    Celltype<-data$Cluster_name
    Group<-data$GROUP
    cellinfo<-data.frame(Sample,Cluster,Celltype,Group)
    scenicOptions <- initializeScenic(org="mgi", dbDir="./SCENIC_database", nCores=10)
    #randomly select 5000 cells for GENIE3
    names<-colnames(count)
    number<-length(names)
    snumber<-sample(number,5000)
    exp_sub<-counts[,snumber]
    genesKept <- geneFiltering(exp_sub, scenicOptions)
    exp_sub_filtered <- exp_sub[genesKept, ]
    runCorrelation(exp_sub_filtered, scenicOptions)
    exp_sub_log <- log2(exp_sub_filtered+1)
    runGenie3(exp_sub_log, scenicOptions)
    ### Build and score the GRN
    exprMat_log <- log2(counts+1)
    scenicOptions@settings$dbs <- scenicOptions@settings$dbs["10kb"] # Toy run settings
    runSCENIC_1_coexNetwork2modules(scenicOptions)
    runSCENIC_2_createRegulons(scenicOptions, coexMethod=c("top5perTarget")) # Toy run settings
    runSCENIC_3_scoreCells(scenicOptions, exprMat_log)
    #results
    coexp<-readRDS("int/1.6_tfModules_asDF.Rds")
    regulonAUC<-loadInt(scenicOptions,"aucell_regulonAUC")
    rss<-calcRSS(AUC=getAUC(regulonAUC),cellAnnotation=cellinfo[colnames(regulonAUC),"Celltype"])
    rssPlot<-plotRSS(rss)
    rssPlot$plot
    pdf("rssPlot_by_sample.pdf",height=5,width=8)
    rssPlot$plot
    dev.off()
    #heatmap
    regulonAUC <- loadInt(scenicOptions, "aucell_regulonAUC")
    regulonAUC <- regulonAUC[onlyNonDuplicatedExtended(rownames(regulonAUC)),]
    regulonActivity_byCellType <- sapply(split(rownames(cellinfo), cellinfo$Celltype),
        function(cells) rowMeans(getAUC(regulonAUC)[,cells]))
    regulonActivity_byCellType_Scaled <- t(scale(t(regulonActivity_byCellType), center = T, scale=T))
    pdf("heatmap_by_cluster.pdf",height=7,width=9)
    ComplexHeatmap::Heatmap(regulonActivity_byCellType_Scaled, name="Regulon activity")
    dev.off()
11. Analysis of differentially expressed gene patterns between clusters.
  a. Differentially expressed genes in different clusters or samples are identified with the FindMarkers package of Seurat.
  b. The differentially expressed genes are then used to perform enrichment analyses, including Gene Set Variation Analysis (GSVA) and Gene Set Enrichment Analysis (GSEA), which are completed with the R packages GSVA (version 1.30.0) and fgsea (version 1.8.0).
    library(tibble)
    library(ggplot2)
    library(cowplot)
    library(Seurat)
    library(fgsea)
    library(msigdbr)
    library(ClusterProfiler)
    #differentiate genes were calculated by FindMarkers
    filelist<-dir("diff_gene_by_group/tumor_diff/")
    ###################
    #use fgsea
    ###################
    #get gmt file
    #the newest gmt files can be downloaded from msigDB.
    gobp_pathway<-gmtPathways("/home/wangrj/wangrj/wangrj/mouse_breasttumor/BD_second/gskb_gmt_files/unfiltered/MousePath_GO_BP_gmt.gmt")
    kegg_pathway<-gmtPathways("/home/wangrj/wangrj/wangrj/mouse_breasttumor/BD_second/gskb_gmt_files/unfiltered/MousePath_Metabolic_KEGG_gmt.gmt")
    #draw picture of each cell type in tumor sample
    draw_gsea_barplot<-function(fl){
     #get ranks from foldchange data
     diffgene<-read.csv(paste0("diff_gene_by_group/tumor_diff/",fl),row.name=1)
     diffgene<-subset(diffgene,p_val_adj<0.05)
     diffgene<-subset(diffgene,p_val<0.05)
     diffgene$gene<-rownames(diffgene)
     diffgene$gene<-toupper(diffgene$gene)
     diffgene<-diffgene[!duplicated(diffgene$gene),]
     rownames(diffgene)<-diffgene$gene
     rownames(diffgene)<-toupper(rownames(diffgene))
     diff_frame<-data.frame(rownames(diffgene),diffgene$avg_logFC)
     ranks<-deframe(diff_frame)
    #gsea analysis
     fgseaRes <- fgsea(pathways = gobp_pathway, stats = ranks,nperm=1000,minSize = 3,maxSize = 500)
     fgdata<-fgseaRes[order(fgseaRes$NES),]
     upgene<-fgdata[(length(rownames(fgdata))-20):length(rownames(fgdata)),]
     downgene<-fgdata[1:20,]
     geneall<-rbind(downgene,upgene)
     #geneall<-subset(fgdata,pval<=0.05)
     geneall$Group<-"NA"
     for(i in 1:length(geneall$ES)){
      if(geneall$NES[i]>0 & geneall$pval[i]<=0.05){
       geneall$Group[i]="Up"
      }
      else if(geneall$NES[i]<0 & geneall$pval[i]<=0.05){
       geneall$Group[i]="Down"
      }
      else if(geneall$pval[i]>0.05){
       geneall$Group[i]="Unsig"
      }
    }
    colnames(geneall)[1]<-"Pathway"
    geneall$Group<-factor(geneall$Group,levels=c("Up","Down","Unsig"))
    geneall$Pathway<-gsub("GO_BP_MM_","",geneall$Pathway)
    geneall$Pathway<-factor(geneall$Pathway,levels=as.character(geneall$Pathway))
    #barplot
    clname<-gsub("_diff_genes.csv","",fl)
    clname<-gsub(" ","_",clname)
    pdfname<-paste0("gsva_plots/tumor_gsva_barplot_gobp_",clname,".pdf")
    titlename=paste0("GSEA_analysis_",clname)
    ggplot(geneall,aes(x=Pathway,y=NES,fill=Group))+geom_bar(stat="identity")+
    scale_fill_manual(values = c("#FF0099","#6699FF","lightgrey"))+
    coord_flip()+guides(fill = "none")+theme_classic()+labs(title=titlename)+
    geom_text(data = subset(geneall, NES>0 & pval<=0.05),aes(x=Pathway, y= 0, label= paste0(Pathway," ")),hjust = "outward")+
    geom_text(data = subset(geneall, NES>0 & pval>0.05),aes(x=Pathway, y= 0, label= paste0(Pathway," ")),color="darkgrey",hjust = "outward")+
    geom_text(data = subset(geneall, NES<0 & pval>0.05),aes(x=Pathway, y= 0, label= paste0(" ",Pathway)),color="darkgrey",hjust = "inward")+
    geom_text(data = subset(geneall, NES<0 & pval<=0.05),aes(x=Pathway, y= 0, label= paste0(" ",Pathway)),hjust = "inward")+
    theme(plot.title=element_text(hjust=0.5,size=18),axis.line.y=element_blank(),axis.ticks.y=element_blank(),axis.text.y=element_blank(),axis.title=element_text(size=15))
    ggsave(pdfname,height=4,width=9)
    }
  c. Use the AddModuleScore package of Seurat to analyze the expression activities of genes involved in important pathways related to antitumor immunity.
    ***Note:*** All the processes are completed on Python (version 2.7.5) and R (version 3.6.3) platforms. Plots are made with the ggplot2 (version 3.3.5), ggpubr (version 0.4.0), pheatmap (version 1.0.12) and ComplexHeatmap (version 2.8.0) packages in R. The scripts below show an example of AddModuleScore analysis of cytotoxic related scores.
    library(Seurat)
    library(cowplot)
    library(future)
    library(Matrix)
    library(ggplot2)
    library(ggpubr)
    library(dplyr)
    library(patchwork)
    lym<-readRDS("lymphocytes_all.rds")
    #genelist are from the gskb database.
    cyt_regulation_score<-list(c("Pnp","Cd1d1","Il12a","Il23a","Ptprc","H2-T23","H2-M3","H2-K1","B2m","Tap1","Tap2","Il12b","P2rx7","Abcb9","H2-D1","Xcl1"))
    cyt_diff_regulation_score<-list(c("Sart1","Hsp90aa1"))
    cyt_score<-list(c("Serpinb9","Gzmb","Ctsc","Ctsh"))
    lym<-AddModuleScore(lym,features=cyt_regulation_score,ctrl=20,name="Cytotoxic_Regulation_Signatures")
    lym<-AddModuleScore(lym,features=cyt_diff_regulation_score,ctrl=5,name="Cytotoxic_Diff_Regulation_Signatures")
    lym<-AddModuleScore(lym,features=cyt_score,ctrl=10,name="Cytotoxic_Signatures")
    Cyto_score<-lym$Cytotoxic_Signatures1
    Cyto_reg_score<-lym$Cytotoxic_Regulation_Signatures1
    Cyto_diff_reg_score<-lym$Cytotoxic_Diff_Regulation_Signatures1
    Group<-lym$GROUP
    Sample<-lym$SAMPLE
    lym$Tissue_days<-paste(lym$TISSUE,lym$DAYS,sep="_")
    Tissue<-lym$Tissue_days
    Cluster<-lym$Cluster_name
    Cluster_detailed<-lym$Cluster_detailed
    data<-data.frame(Group,Cyto_score,Cyto_reg_score,Cyto_diff_reg_score,Sample,Tissue,Cluster,Cluster_detailed)
    ggplot(subset(data,Cluster=="Cd8+ T cells"),aes(x=Group,y=Cyto_score,fill=Group))+geom_boxplot()+
    theme_classic()+facet_wrap(∼Tissue)+
    #stat_compare_means(map_signif_level=T,label.x.npc="middle")+
    theme(text=element_text(size=15))
    ggsave("Cyto_score_graph_CD8_no_label.pdf",height=5,width=6)
12. Cell-cell communication analysis. This procedure is completed with the R package (version 1.0.0).^5^
  a. Choose five cell types that played important roles in the antitumor immunity for communication analysis.
  b. Calculate and compare the communication numbers and strengths between the normal and Sephin1 groups in different tissues.
    library(CellChat)
    library(ComplexHeatmap)
    library(ggplot2)
    mdata<-readRDS("mouse_all_data_20211017.rds")
    #celltype: CD4, CD8, NK, macrophage, DC
    celltypes<-c("Cd4+ T cells","NK cells","Cd8+ T cells","Macrophages","DCs")
    msub<-subset(mdata,Cluster_name %in% celltypes)
    saveRDS(msub,"mouse_Tcell_NK_Mac_DC.rds")
    cellchat_cal<-function(subdata){
     m_cellchat<-createCellChat(subdata,group.by="Cluster_name")
     groupsize<-as.numeric(table(m_cellchat@idents))
     ccdb<-CellChatDB.mouse
     showDatabaseCategory(ccdb)
     m_cellchat@DB<-ccdb
     m_cellchat<-subsetData(m_cellchat)
     m_cellchat<-identifyOverExpressedGenes(m_cellchat)
     m_cellchat<-identifyOverExpressedInteractions(m_cellchat)
     m_cellchat<-projectData(m_cellchat,PPI.mouse)
     m_cellchat<-computeCommunProb(m_cellchat,raw.use=F,population.size=T)
     m_cellchat<-filterCommunication(m_cellchat,min.cells=10)
     df.net<-subsetCommunication(m_cellchat)
     m_cellchat<-computeCommunProbPathway(m_cellchat)
     df.netp<-subsetCommunication(m_cellchat,slot.name="netP")
     m_cellchat<-aggregateNet(m_cellchat)
     groupSize<-as.numeric(table(m_cellchat@idents))
     return(m_cellchat)
    }
    #calculate cellchat results of every sample type
    samlist<-unique(msub$SAMPLE)
    msub_cc<-list()
    for(sa in samlist){
     sub<-subset(msub,SAMPLE==sa)
     cchat<-cellchat_cal(sub)
     msub_cc<-c(msub_cc,list(cchat))
    }
    names_title<-c("Normal_B_d0","Sephin1_B_d0","Normal_B_d15","Sephin1_B_d15","Normal_T_d15","Sephin1_T_d15")
    names(msub_cc)<-names_title
    saveRDS(msub_cc,"mouse_Tcell_NK_Mac_DC_cellchat_by_sample.rds")
    msub_cc<-readRDS("mouse_Tcell_NK_Mac_DC_cellchat_by_sample.rds")
    #netgraph of normal and sephin1 group
    tsub<-c(msub_cc[[5]],msub_cc[[6]])
    #weight.max <- getMaxWeight(tsub, attribute = c("idents","weight"))
    weight.max<-getMaxWeight(msub_cc, attribute = c("idents","weight"))
    pdf("netgraph_five_celltypes.pdf",height=5,width=6)
    for(i in 1:6){
     print(netVisual_circle(msub_cc[[i]]@net$weight, weight.scale = T,
       label.edge= F, edge.weight.max = weight.max[2],
       edge.width.max = 20, arrow.size=0.05,arrow.width=0.1,
       title.name = paste0("Interaction weight - ", names(msub_cc)[i])))
    }
    dev.off()
    #different types of graph of differentiated pathways
    cellchat <- mergeCellChat(msub_cc, add.names = names(msub_cc))
    titlenames<-c("Day0_Blood","Day15_Blood","Day15_Tumor")
    #netgraph
    pdf("differentiated_netgraph_five_celltypes.pdf",height=5,width=6)
    for(i in 1:3){
     num1<-2∗i-1
     num2<-2∗i
     tname<-titlenames[i]
     print(netVisual_diffInteraction(cellchat, comparison=c(num1,num2),weight.scale = T,
        measure = "weight",title.name=tname))
    }
    dev.off()
    #bubbleplot of selected cell types
    siglist<-c("APP","MIF","MHC-I","SEMA4")
    pdf("bubble_plot_of_selected_celltypes_and_selected_pathways_0718.pdf",height=6,width=8)
    for(i in 1:3){
     tname<-titlenames[i]
     tname1<-paste0("Increased signaling in ",tname,", NK & Cd8")
     tname2<-paste0("Decreased signaling in ",tname,", NK & Cd8")
     print(netVisual_bubble(cellchat,signaling=siglist,source=c("NK cells","Cd8+ T cells"),target=c("NK cells","Cd8+ T cells"),comparison = c(2∗i-1, 2∗i), max.dataset = 2∗i,
        title.name = tname1, remove.isolate = F,angle.x=45))
     print(netVisual_bubble(cellchat,signaling=siglist,source=c("NK cells","Cd8+ T cells"),target=c("NK cells","Cd8+ T cells"),comparison = c(2∗i-1, 2∗i), max.dataset = 2∗i-1,
        title.name = tname2, remove.isolate = F,angle.x=45))
    }
    dev.off()
    bubble_data<-list()
    for(i in 1:3){
     tname<-titlenames[i]
     tname1<-paste0("Increased signaling in ",tname)
     tname2<-paste0("Decreased signaling in ",tname)
     bubble_data<-c(bubble_data,list(netVisual_bubble(cellchat,source=c("NK cells","Cd8+ T cells"),target=c("NK cells","Cd8+ T cells"),comparison = c(2∗i-1, 2∗i),
        title.name = tname1, remove.isolate = T,return.data=T)$communication))
    }
    names(bubble_data)<-c("Day0_Blood","Day15_Blood","Day15_Tumor")
    #calculate the strength of each pathway and compare
    #strength=prob1+prob2+....
    result_list<-list()
    result_all<-c()
    rnames<-c("Day0_Blood","Day15_Blood","Day15_Tumor")
    for(i in 1:3){
     result<-c()
     sub1<-bubble_data[[i]]
     samplename<-unique(sub1$dataset)
     pathway<-unique(sub1$pathway_name)
     for(sn in samplename){
      grlist<-str_split(sn,"_",simplify=T)
      gr<-grlist[1]
      for(pt in pathway){
       sub2<-subset(sub1,pathway_name==pt & dataset==sn)
       str_each<-sum(sub2$prob)
       num_each<-length(sub2$prob)
       deach<-data.frame(sn,pt,str_each,num_each,rnames[i],gr)
       result<-rbind(result,deach)
      }
     }
    colnames(result)<-c("Sample","Pathway","Strength","Number","Tissue","Group")
    result_list<-c(result_list,list(result))
    result_all<-rbind(result_all,result)
    }
    ggplot(result_all,aes(x=Pathway,y=Strength,fill=Group))+
     geom_bar(stat="identity",position="dodge")+facet_wrap(∼Tissue)+theme_bw()+
     theme(text=element_text(size=15),axis.text.x=element_text(angle=90,hjust=1))
    #heatmap making, final heatmap was drawn by ComplexHeatmap in personal desktop.
    pathway_name<-unique(result_all$Pathway)
    result_all<-data.table(result_all)
    result_map<-c()
    sample_name<-c("Normal_B_d0","Sephin1_B_d0","Normal_B_d15","Sephin1_B_d15","Normal_T_d15","Sephin1_T_d15")
    for(pn in pathway_name){
     deach<-c()
     for(sn in sample_name){
      sub1<-subset(result_all,Sample==sn)
      if(pn %in% sub1$Pathway){
       sub2<-subset(sub1,Pathway==pn)
       str_num<-sub2$Strength[1]
      }
      else{
       str_num<-0
      }
      deach<-c(deach,str_num)
     }
    result_map<-rbind(result_map,deach)
    }
    colnames(result_map)<-sample_name
    rownames(result_map)<-pathway_name
    acol<-rep(c("Day0_Blood","Day15_Blood","Day15_Tumor"),each=2)
    acol<-data.frame(acol)
    rownames(acol)<-colnames(result_map)
    colnames(acol)<-"Tissue"
    library(scales)
    library(dplyr)
    result_map1<-log10(100+result_map)
    result_new<-scale(result_map1)
    result2<-scale(result_map)
    p1=pheatmap(result_map,cluster_rows = F,cluster_cols = F,scale="row",gaps_col=c(2,4),
     labels_col=rep(c("Normal","Sephin1"),3),angle_col=0,annotation_col=acol)
    anno<-apply(result_map,1,sum)
    p2=rowAnnotation(foo=anno_barplot(anno))
    siglist_mac<-c("SPP1","THBS","TGFb","APP","CD45","TNF","IL2")
    pdf("bubble_plot_of_Cd4_mac_and_selected_pathways_by_heatmap.pdf",height=9,width=6)
    for(i in 1:3){
     tname<-titlenames[i]
     tname1<-paste0("Increased signaling in ",tname,", Mac & Cd4")
     tname2<-paste0("Decreased signaling in ",tname, ", Mac & Cd4")
     print(netVisual_bubble(cellchat,source=c("Macrophages","Cd4+ T cells"),target=c("Macrophages","Cd4+ T cells"),comparison = c(2∗i-1, 2∗i), max.dataset = 2∗i,
        title.name = tname1, remove.isolate = F,signaling=siglist_mac))
    print(netVisual_bubble(cellchat,source=c("Macrophages","Cd4+ T cells"),target=c("Macrophages","Cd4+ T cells"),comparison = c(2∗i-1, 2∗i), max.dataset = 2∗i-1,
        title.name = tname2, remove.isolate = F,signaling=siglist_mac))
    }
    dev.off()

### Single-cell result confirmation by FACS analysis and *in vitro* experiments

**Timing: 2 weeks**

This procedure describes the FACS analysis and *in vitro* experiments in accordance with the single-cell analysis results.

13. FACS analysis of tumor microenvironment. Tumor tissues are dissociated by the above-mentioned procedure. The weight of each tumor tissue should be measured before this procedure.
  a. Stimulate the cells with Cell Activation Cocktail with Brefeldin A (BioLegend, 423303) at a cell density of approximately 5 × 10ˆ6 cells/mL.
    ***Note:*** This step is performed to stimulate the secretion of IFNγ. The volume ratio of the cocktail and the cell suspension was 1:500.
  b. After stimulating at 37°C for 4 h, centrifuge the cells at 400 × g for 7 min and discard the supernatant.
  c. Incubate the cells with appropriate surface markers, including CD45 (Thermo Fisher, 12-0451-83; BioLegend, 103105; BioLegend, 157607), CD3E (BD Pharmingen, 553064), CD4 (BioLegend, 100548), CD8A (Thermo Fisher, 25-0081-81), NK1.1 (Thermo Fisher, 48-5941-80), FOXP3 (BioLegend, 126419), PD-1 (Thermo Fisher, 17-9985-82), CD11b (BioLegend, 101215), F4/80 (BioLegend, 123125), TCRβ (BioLegend, 109205) and reagents from a LIVE/DEAD Viability Kit (ThermoFisher, L34994/L34963) for 30 min. The volume ratio of the LIVE/DEAD Viability Kit to the cell suspension is 1:1000, and the ratio of other antibodies to the cell suspension is 1:200. The concentration of cell suspension is no more than $10^{8}$/mL.
  d. Centrifuge the cells at 1500 rpm for 5 min and wash with PBS once.
  e. Fix and permeabilize cells with Cytofix/Cytoperm Kit (554714, BD Pharmingen) as instructed by manufacturer (https://www.bdbiosciences.com/en-us/products/reagents/flow-cytometry-reagents/research-reagents/buffers-and-supporting-reagents-ruo/fixation-permeabilization-kit.554714) for 30 min at 4°C.
  f. Centrifuge the cells at 1500 rpm for 5 min, and then discard the supernatant completely.
  g. Incubate the cells with a mouse IFNG-specific antibody (BioLegend, 505806) for 30 min at 4°C. The ratio of the antibody to the cell suspension is 1:200. The concentration of cell suspension is no more than $10^{8}$/mL.
  h. Wash and resuspend the cells with BD Perm/Wash buffer from the Cytofix/Cytoperm Kit according to the manufacturer’s instructions (https://www.bdbiosciences.com/en-us/products/reagents/flow-cytometry-reagents/research-reagents/buffers-and-supporting-reagents-ruo/fixation-permeabilization-kit.554714).
  i. Perform FACS analysis with a CytoFLEX LX from Beckman Coulter (Figure 4).

**Figure 4:**
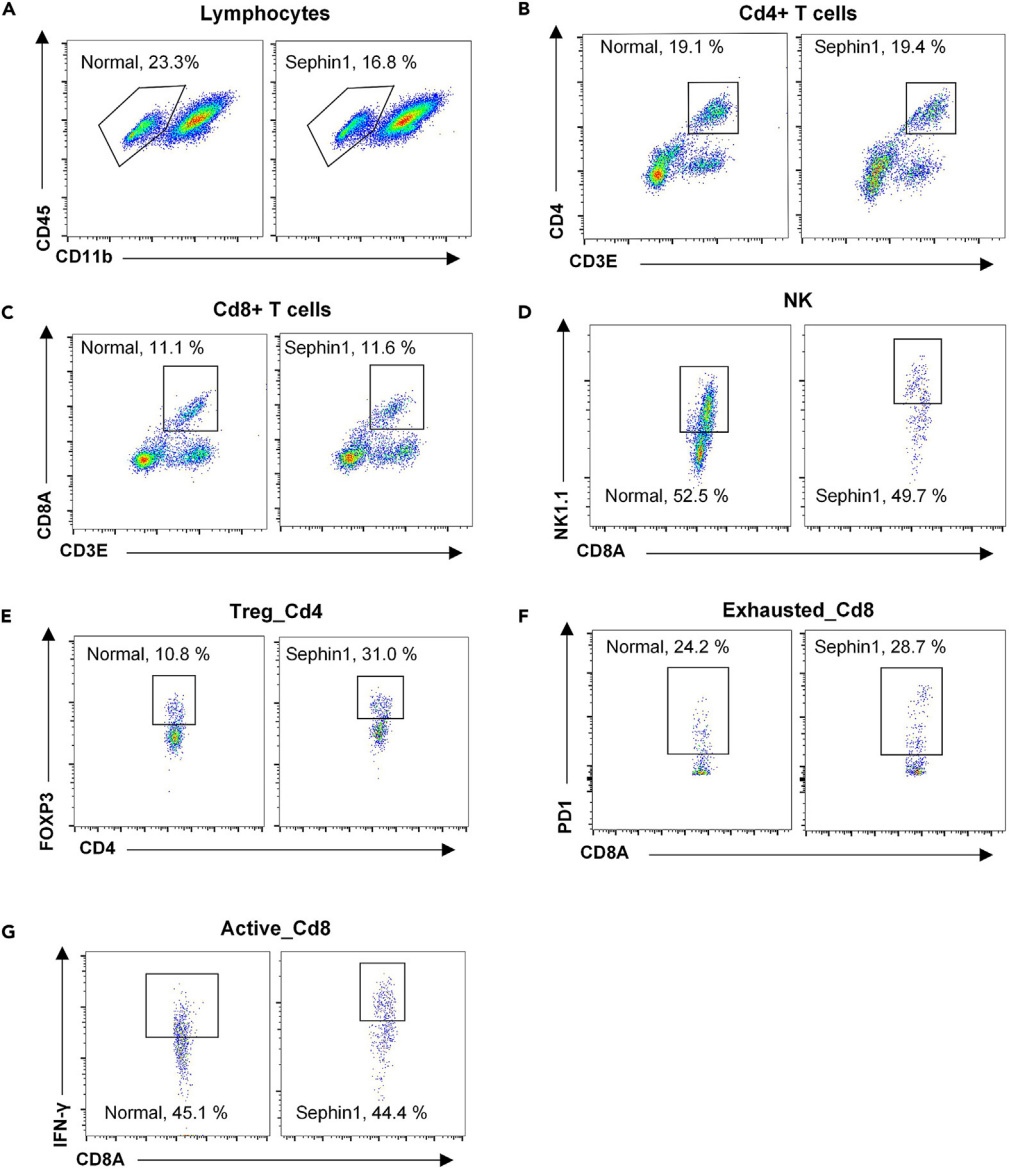
FACS gating strategy for cell sorting^1^ Firstly gating the CD45+ immune cells (A), then gating Cd4+ and Cd8+ T cells by CD3E & CD4/CD8A (B & C), NK cells by NK1.1 (D), Cd4+ regular T cells by CD4 and FOXP3 (E), exhausted Cd8+ T cells by CD8A and PD1 (F) and active Cd8+ T cells by CD8A and IFN-γ (G).
14. *In vitro* analysis of the effect of Sephin1 on mouse CD8+ T cells. This procedure is used to confirm the suppression effect of Sephin1 on CD8+ T cells *in vitro*.
  a. First prepare a round-bottom 96-well plate with 100 μL PBS supplemented with 1 μg/mL anti-mouse CD3ε (BioXCell, BE0001-1-5MG) and anti-mouse CD28 (BioXCell, BE0015-1-5MG) the day before CD8+ T-cell isolation. Discard the solution completely before use.
  b. Isolate the CD8+ T cells from the spleen tissue of 6–8 week-male C57BL/6 mice with a MojoSort Mouse CD8 T Cell Isolation Kit (BioLegend, 480035) according to the standard protocol.
  c. After separation, firstly incubate the isolated CD8+ T cells with reagents from a CFSE Cell Division Tracker Kit (BioLegend, 423801) according to the manufacturer’s instructions (https://www.biolegend.com/en-us/products/cfse-cell-division-tracker-kit-9396) and then resuspend the cells in RPMI 1640 medium (Gibco, 11875093) supplemented with 10% FBS (Biological Industries, 04-001-1A) and 1% Pen Strep (Gibco, 15140122).
  d. Add mouse IL2 (Novoprotein, CK24) and IL7 (Novoprotein, CC73) into the medium for a concentration of 20 ng/mL to stimulate the proliferation of T cells. The cell concentration is between 5 $\times 10^{5}$ /mL to 10^6^/mL.
  e. Incubate the isolated CD8+ T cells in the precoated 96-well plates (step 14. a.) for 72 h.
  f. Centrifuge at 400 × g for 5 min to collect the cells. Remove the supernatant completely.
  g. Stain with a LIVE/DEAD Fixable Violet Dead Cell Stain Kit (Invitrogen, L34963), PerCP/Cyanine 5.5-conjugated anti-mouse CD8a antibody (BioLegend, 100733) and PE-conjugated anti-mouse IFN-γ antibody (BioLegend, 163503) as described above.
  h. Analyze the prepared cells on a CytoFLEX LX machine from Beckman Coulter.

## Expected outcomes

**Figure 1:**
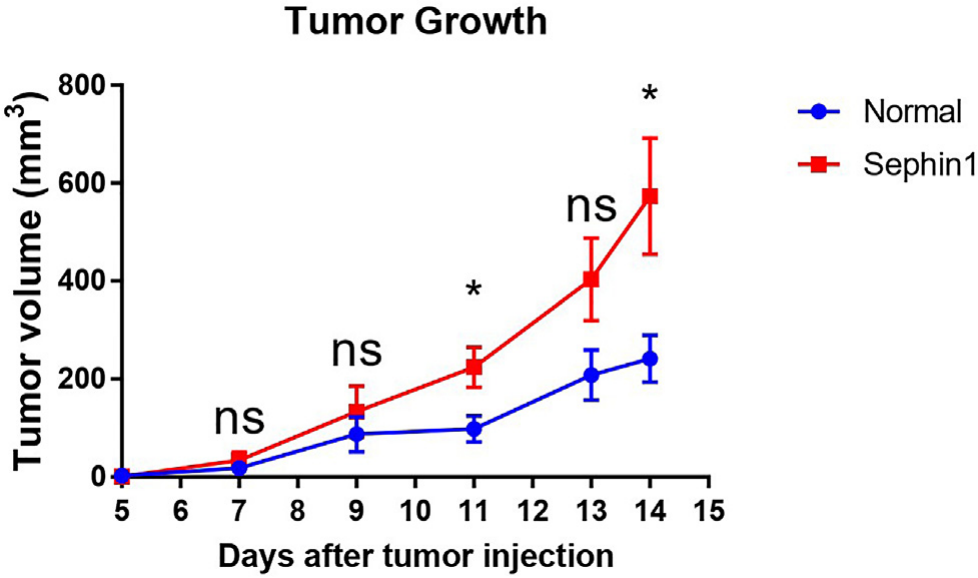
Tumor growth curve in the normal and Sephin1 group^1^ Data are represented as mean ± SEM.

**Figure 2:**
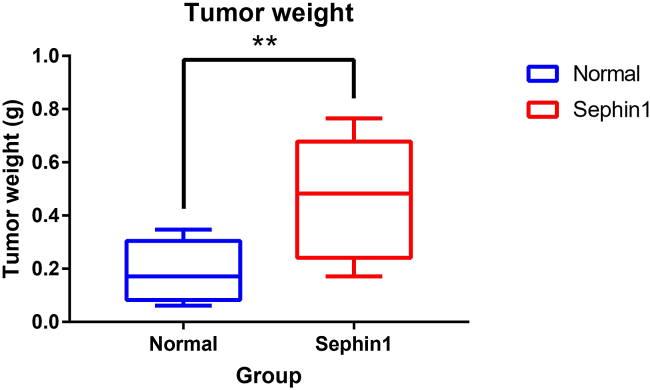
Tumor weight in the normal and Sephin1 group^1^ Data are represented as mean ± SEM.

**Figure 3:**
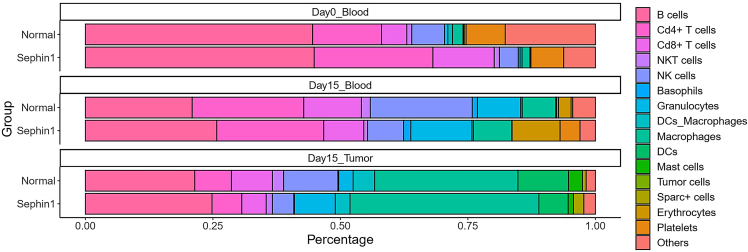
Cell type composition in different samples^1^

This protocol provides a detailed protocol including mouse model construction, cell isolation and single-cell data analysis. After 14–15 days tumor growth, the tumor volume in the control group is around 200–300 mm^3^, and in the Sephin1 treated group is around 600 mm^3^ (Figure 1). The average tumor weight at that time point in the control group is around 0.2 g, and around 0.5 g in the Sephin1 group (Figure 2). About 10^6^ PBMCs can be collected from 100-200 μl of blood sample. The tumor immune cells are collected through FACS sorting, and live CD45+ cells make up about 1–2% of cells of dissociated tumor. The main immune cell types from all samples include B cells, T cells, NK cells, NKT cells, macrophages, and various other cell types (Figure 3). In addition, the total number of immune cells in each mouse spleen is about 5 $\times 10^{7}$, and about 10% CD8+ T cells can be isolated by the MojoSort Mouse CD8 T Cell Isolation Kit.

## Quantification and statistical analysis

All statistical analyses were conducted using GraphPad Prism 7 (La Jolla, CA, USA) or R (version 3.6.3). Statistic differences between groups were calculated with different methods depended on the data characteristics, including unpaired two-tailed Student’s t-test, Chi square test, or Wilcoxon test (annotated in the figure legends). Statistical parameters are either shown with exact p values or as: ∗: p < 0.05, ∗∗: p < 0.01, ∗∗∗: p < 0.001, ∗∗∗∗: p < 0.0001.

## Limitations

In the mouse model construction process, this protocol concentrates on the subcutaneous tumor construction from the melanoma cell line B16F1. Other tumor types and tumor model construction methods are not used in this protocol, which limits the application scope of this protocol.

In the single-cell library construction procedure, only the expression library and TCR enrichment library are constructed. The BCR enrichment library may also be useful and have important functions in the antitumor immune process.

In addition, this protocol only explores the composition of immune cells in the tumor microenvironment and peripheral blood. Immune cells in the lymph glands and spleens can also play a key role in the antitumor immunity. Change in the tumor composition over time is also important to explore the function of Sephin1, but not fully studied in this research.

## Troubleshooting

### Problem 1

When making the Sephin1 solution, the powder cannot be fully dissolved in the solvent.

### Potential solution

• When dissolving the Sephin1 powder, first, dissolve the powder in the proper amount of DMSO, and then diluting the Sephin1-DMSO into the proper amount of PBS. After that, incubate the solution in 37°C to maximize the solubility. The solution needs to be mixed thoroughly every time before use.

### Problem 2

In the tumor sample, there is a low fraction of living immune cells, which makes it difficult for their isolation.

### Potential solution

• The mouse should be killed immediately before the tumor tissue collection to shorten the wait time. When digesting the single-cell suspension from tumor tissue, make sure that the standard dissociation protocol from the dissociation kit is carried out exactly as the protocol described. After dissociation, the cell suspension should be filtered by 100 μm cell strainer firstly to remove aggregated cells.

### Problem 3

The expression data is large, and the data process can be time consuming with large memory requirements.

### Potential solution

• Use the server with multithread and large RAM. The server with threads more than 32 and RAM larger than 500 GB is preferred. Better performance of the server can shorten the running time of reads mapping and data integration process.

### Problem 4

Some samples may have erythrocyte contamination and influence the expression results, especially in PBMC samples.

### Potential solution

• The best way is to remove the red blood cells as thoughly as possible during the sample preparation process. If there are still remaining red blood cells(RBCs) during single-cell data analysis, several steps should be done before analysis. First, after the integration step, expression data should be scaled by adding the vars.to.regress parameter(mouse.data<-ScaleData(mouse.data,vars.to.regress = "percent.mt")) to minimize the influence of RBCs-related genes. Second, clusters identified as red blood cells should be removed from the data. The remaining cells are used for further analysis.

### Problem 5

There might be some conflicts between the operation system and different versions of R package, resulting in the installation failure of some packages.

### Potential solution

• Use Conda to install the needed environment of specific R or Python version. For example, if an R package cannot be installed properly by the “install.packages” function, in most cased, it can be installed by: conda install -c conda-forge r-{package-name}.

## Resource availability

### Lead contact

Further information and requests for resources and reagents should be directed to and will be fulfilled by the lead contact, Xiangyin Kong (xykong@sibs.ac.cn).

### Materials availability

No new unique reagents were generated for this protocol.

## Data Availability

The raw and processed data related to this protocol are deposited at GEO: GSE220656. Code related to this protocol is deposited at the website: https://github.com/LisaWang2022/B16F1-mouse-single-cell-analysis.
